# Salivary inflammatory mediators as biomarkers for oral mucositis and oral mucosal dryness in cancer patients: A pilot study

**DOI:** 10.1371/journal.pone.0267092

**Published:** 2022-04-27

**Authors:** Anna Kiyomi, Kensuke Yoshida, Chie Arai, Risa Usuki, Kyosuke Yamazaki, Naoto Hoshino, Akira Kurokawa, Shinobu Imai, Naoto Suzuki, Akira Toyama, Munetoshi Sugiura

**Affiliations:** 1 Department of Drug Safety and Risk Management, School of Pharmacy, Tokyo University of Pharmacy and Life Sciences, Tokyo, Japan; 2 Division of Oral and Maxillofacial Surgery, Faculty of Dentistry & Graduate School of Medical and Dental Sciences, Niigata University, Niigata, Japan; 3 Division of Hospital Pharmacy, Niigata University Medical and Dental Hospital, Niigata, Japan; University of Pisa, ITALY

## Abstract

Oral mucositis (OM) is a common side effect in patients with cancer receiving chemotherapy and radiotherapy; however, no salivary mediator is known to be associated with OM. We aimed to determine candidate salivary inflammatory mediators potentially associated with OM in patients with cancer. To this end, we compared the relationships between OM grade, oral mucosal dryness, and inflammatory mediators (Interleukin (IL)-1β, IL-6, IL-10, IL-12p70, tumor necrosis factor (TNF), prostaglandin E2, and vascular endothelial growth factor) in patients with cancer and in healthy volunteers (HV). We collected saliva samples from 18 patients with cancer according to the following schedule: 1) within 14 days of treatment initiation, 2) within 3 days of OM occurrence, 3) when OM was improved or got worsened, and 4) within 7 days after chemotherapy completion. The oral care support team determined the OM grade at each sample collection point based on CTCAE version 5.0. Salivary inflammatory mediator concentrations were detected using cytometric bead array or enzyme-linked immunoassay. We compared oral mucosal dryness in pre- and post-index patients with cancer to that in HV (n = 33) using an oral moisture-checking device. Fourteen of eighteen patients experienced OM (four, grade 3 OM; four, grade 2 OM; six, grade 1 OM). IL-6, IL-10, and TNF salivary concentrations were significantly increased in the post-index group compared to those in the pre-index group (*p* = 0.0002, *p* = 0.0364, and *p* = 0.0160, respectively). Additionally, salivary IL-6, IL-10, and TNF levels were significantly higher in the post-index group than in the HV group (*p* < 0.0001, *p* < 0.05, and *p* < 0.05, respectively). Significant positive correlations were observed between OM grade and salivary IL-6, IL-10, and TNF levels (*p* = 0.0004, r = 0.4939; *p* = 0.0171, r = 0.3394; and *p* = 0007, r = 0.4662, respectively). Oral mucosal dryness was significantly higher in the HV than in the pre- and post-index groups (*p* < 0.001). Our findings suggest that salivary IL-6, IL-10, and TNF levels may be used as biomarkers for OM occurrence and grade in patients with cancer. Furthermore, monitoring oral mucosal dryness and managing oral hygiene before cancer treatment is essential.

## Introduction

Oral mucositis (OM) is an inflammatory condition of the oral mucosa characterized by painful erythematous and ulcerative lesions in the oral cavity [1]. OM occurs as a common side effect in patients with cancer receiving chemotherapy (CTx) and radiotherapy (RT), which can be severe and impede the quality of life [2–4]. More than 75% of the patients with leukemia undergoing CTx and bone marrow transplantation (BMT) experience OM [5, 6], and > 40% of those receiving RT experience ≥ grade 3 OM [7, 8].

Regarding the pathobiology of OM, free radicals activated by CTx and RT are reportedly responsible for the upregulation of certain genes via the NF-κB pathway, leading to the excessive production of inflammatory mediators such as interleukin (IL)-1b, IL-6, tumor necrosis factor (TNF), and prostaglandin E2 (PGE2) [9]. A previous study showed that inflammatory mediators were increased in the saliva of patients with head and neck cancer [10]. Although these mediators may be candidates for predicting OM and its severity in patients with cancer, changes in salivary inflammatory mediator levels before and after cancer treatment (TR) remain unknown. Furthermore, no reports have investigated how pre-existing factors, such as the degree of mouth dryness or the patient general oral health, may affect inflammatory mediators in saliva.

Oral mucosal dryness is a highly prevalent and significant symptom in patients with advanced progressive cancers [11]. Thus, basic oral care, including tooth brushing, flossing, mouth rinses, and preventive medication that aims to maintain oral mucosal moisture, is recommended for patients with cancer [12]. Oral dryness should be measured objectively; however, few studies have investigated oral dryness and OM in patients with cancer.

We aimed to to determine a candidate variable that was potentially associated with OM in patients with leukemia or head and neck carcinoma because these cancers are associated with high rates of OM, and have relatively fixed TR periods. Additionally, we analyzed the relationship of OM and oral mucosal dryness with salivary inflammatory mediators in patients with cancer compared to those of healthy volunteers (HV).

## Patients and methods

### Sample collection in patients with cancer

This study was approved by the Ethics Committees of the Niigata University Medical and Dental Hospital (approval no. 2019–0449) and the Tokyo University of Pharmacy and Life Sciences (approval no. 19–25 and 20–3). Informed consent was obtained from all the 20 patients before enrollment. Two patients were excluded because of pre-existing OM and one patient declined the post index sampling. The final 18 participants were patients with cancer aged 21–79 years (10 men, 8 women), with leukemia (n = 12) or head and neck carcinoma (n = 6) who were hospitalized for receiving CT or RT at the Niigata University Medical and Dental Hospital between June 2020 and October 2021 (Table 1).

**Table 1:** Patient characteristics.

Patient number	Sex	Age (years)	Height (m)	Weight (kg)	BMI (kg/m^2^)	Cancer type	Transplant	Chemotherapy	Radiotherapy (total Gy)	Maximum OM grade
1	Male	32	1.63	58.3	22.1	Ph^+^ ALL	PBSCT	FLU/ivBU/CY	3	0
2	Male	59	1.71	63.1	21.5	AML	urBMT	FLU/MEL/MTX	-	1
3	Male	58	1.66	68.8	24.8	AML	urBMT	FLU/MEL/MTX	-	3
4	Male	34	1.70	55.0	19.0	AML	rPB	ivBU/CY/MTX	-	2
5	Female	42	1.56	47.2	19.3	LBL	CBT	FLU/MEL/MTX	3	1
6	Female	51	1.56	38.2	15.6	DLBLC	CBT	FLU/MEL/MTX	-	3
7	Male	43	1.78	76.8	24.2	MDS	-	ivBU/CY/MTX	-	1
8	Female	33	1.59	55.2	21.8	AML	CBT	FLU/MEL/MTX	-	0
9	Male	21	1.81	67.2	20.6	ALL	rPB	FLU/MEL/MTX	-	1
10	Male	46	1.65	82.7	30.2	AML	PBSCT	ivBU/CY/MTX	-	0
11	Female	32	1.61	51.6	20.0	AML	PBSCT	ivBU/CY/MTX	-	0
12	Female	41	1.62	59.6	22.8	AML	-	ivBU/CY/MTX	-	2
13	Male	64	1.74	91.7	30.5	LGC	-	C-mab	44	3
14	Female	78	1.39	35.9	18.7	LGC	-	-	70	2
15	Female	79	1.48	45.1	20.6	UGC	-	C-mab	46	3
16	Male	73	1.58	54.1	21.6	TC	-	CDDP	60	1
17	Male	42	1.59	57.0	22.5	TC	-	CDDP	-	2
18	Female	33	1.48	48.0	22.0	TC	-	CDDP	-	1

Abbreviations: BMI, body mass index; Ph^+^, Philadelphia chromosome-positive; ALL, acute lymphoblastic leukemia; AML, acute myelocytic leukemia; LBL, lymphoblastic lymphoma; DCBLC, diffuse large B-cell lymphoma; LGC, lower gingival carcinoma; UGC, upper gingival carcinoma; TC, tongue cancer; MDS, myelodysplastic syndrome; PBSCT, peripheral blood stem cell transplantation; urBMT, unrelated bone marrow transplant; rPB, related peripheral blood transplantation; CBT, cord blood transplantation; FLU, fludarabine; ivBU, intravenous busulfan; CY, cyclophosphamide; MEL, melphalan; MTX, methotrexate; C-mab, cetuximab; CDDP, cisplatin; OM, oral mucositis.

**Fig 1:**
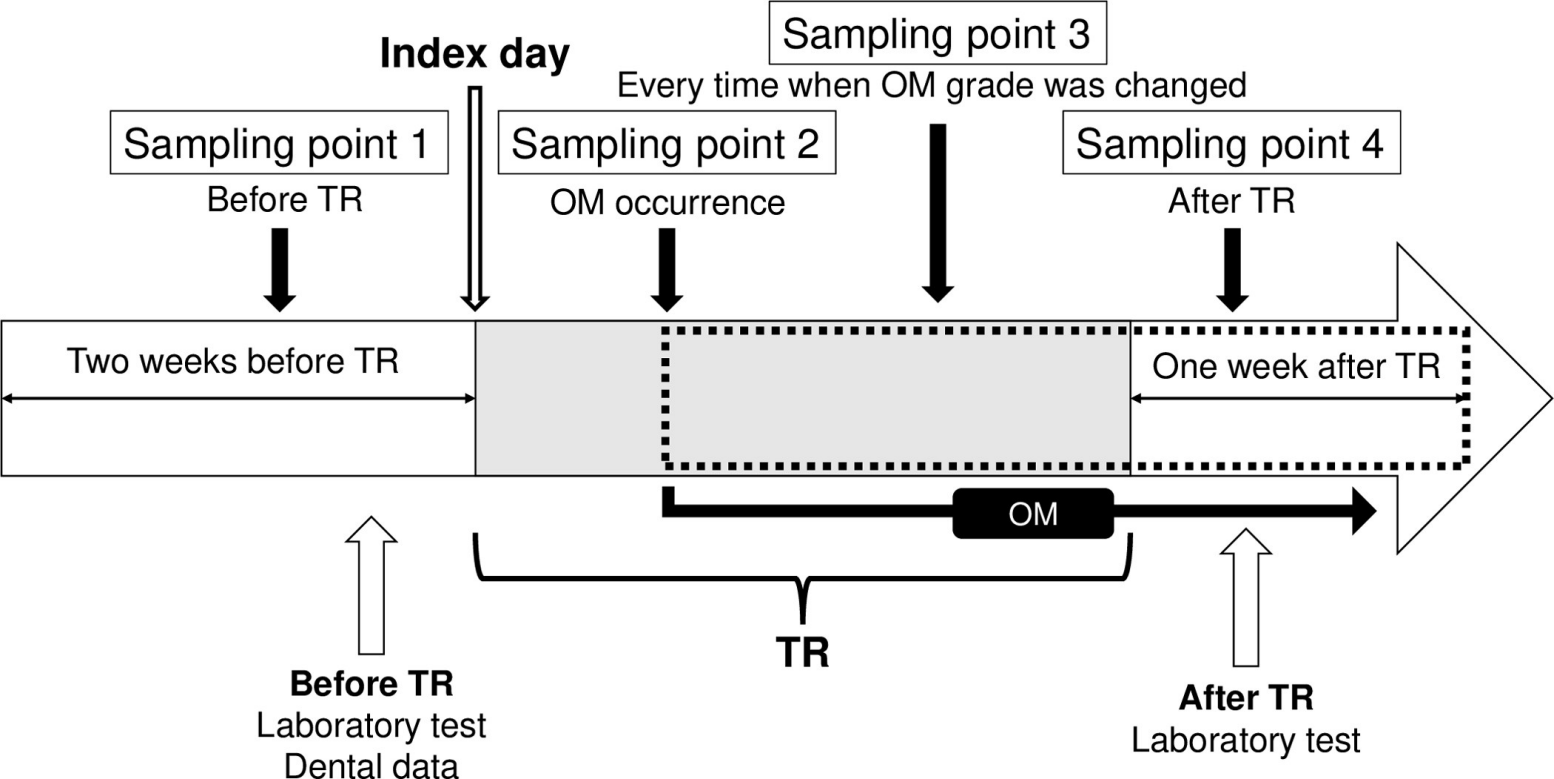
Sampling schedule. The saliva is collected at the sampling points 1–4. Sampling point 1: before TR, sampling point 2: when OM occurred, sampling point 3: when OM grade changed, and sampling point 4: after TR. Abbreviations: TR, treatment; OM, oral mucositis.

We collected ≥ 0.1 mL of saliva using the Saliva Collection Aid (SalivaBio, California, USA), i.e., the drooling method or Salimetrics Oral Swab (Salimetrics, California, USA), i.e., the swab method, at several points (Fig 1). Saliva collection was performed using the non-stimulated method [13] since recruited individuals were patients who would experience OM. Sampling was conducted according to the manufacturer’s instructions, which stated that neither eating of food nor brushing of teeth was to be done within 1 h before sampling; gargling with water and removal of lip makeup was done 15 min in advance, and patient was to rest for 5 minutes before sampling. Saliva samples were collected according to the following schedule: 1) within 14 days of TR initiation, 2) within 3 days of an OM event, 3) if and when the OM event was improved or got. worsened, and 4) within 7 days after TR completion. Sampling was conducted during routine clinical practice; two samples were collected in the morning and 50 were collected in the afternoon. Samples were stored immediately at -80°C. Sampling was omitted when a patient had no OM occurrence. The index date was defined as the first day of TR. Oral mucosal dryness was measured by an oral health care support team of dentists, dental hygienists, nurses, and pharmacists using an oral moisture-checking device (Mucus®; Life Co., Ltd., Saitama, Japan) at each sampling point following the manufacturer’s instructions. Oral mucosal dryness was measured three times in each subject and the median was reported. No specific unit of measurement was used for measuring oral mucosal dryness, and the numeric value provided by the device was used.

The oral care support team recorded the plaque control record (PCR), bleeding on probing (BOP), pocket depth, and tooth mobility before TR (Table 2) and determined the OM grade at each sample collection point based on CTCAE version 5.0. The condition without OM was described as “grade 0”. Hematological and biochemical laboratory data were collected the day before TR, the day of an OM event, and the day after TR (S1 Table).

**Table 2:** Dental data of patients.

*Patient number*	*PCR (%)*	*BOP (%)*		*Tooth mobility^a^ (maximum score)*	*Total tooth number*	*Periodontitis severity^b^*
1	16.1	11	2	0	28	Moderate
2	9.8	10	4	1	28	Moderate
3	-	10	15	0	31	Severe
4	11.6	47	1	0	28	Moderate
5	4.5	5	5	0	28	Moderate
6	1.1	4	5	0	24	Severe
7	16.2	11	12	0	20	Severe
8	38.5	60	10	2	13	Severe
9	61	20	11	3	25	Severe
10	4.5	9	3	0	22	Moderate
11	20.5	35	9	1	28	Moderate
12	26.2	-	-	-	-	-
13	4.2	-	-	-	-	-
14	5.4	-	-	-	-	-
15	5.8	-	-	-	-	-
16	4.7	16	2	0	28	Moderate
17	7.3	10	9	1	31	Moderate
18	62.5	5	9	2	18	Moderate

All assessments are conducted by dentists before treatment.

^a^Tooth mobility was scored as follows: 0, no physiologic movement when force was applied; 1, mobility < 1 mm; 2, mobility of 1–2 mm (horizontal); 3, mobility both horizontal and vertical.

^b^Periodontitis severity is described as the maximum severity based on the periodontal pocket depth as follows: slight, < 4 mm; moderate, 4–6 mm; severe, > 6 mm. Abbreviations: PCR, plaque control record; BOP, bleeding on probing.

All patients were administered a sodium gualenate hydrate oral gargle, dimethyl isopropylazulene oral ointment, and Episil®, a bio-adhesive barrier-forming oral liquid gel, as preventive medication for OM. Patients completed a questionnaire on dietary intake times, sleep times, caffeine intake within an hour, total parenteral nutrition (TPN), and dental treatment within 24 hours before sampling (S2 Table). The questionnaire was administered the same time as sampling to ensure that they followed the protocol and more information was added on personal habits, in order to understand the background differences among patients.

### Sample collection in HV

Volunteers with stomatitis or oral hemorrhage and those under medication were excluded. A total of 33 informed and consenting HV aged 18–65 years (17 men, 16 women) were recruited (S3 Table). Oral mucosal dryness was measured using Mucus^®^ following the previously described methods. Non-stimulated saliva from HV was collected using the drooling method, in order to match the collection method of more than 90% of saliva samples from patients with cancer; which had been collected using this method. The saliva was kept at -80°C until inflammatory mediators were measured. No volunteer had a history of dental treatment within 1 h of sample collection, caffeine intake within 1 h of sample collection, or smoking. Seven samples were collected in the morning and 26 were collected in the afternoon.

### Measurement of inflammatory mediators

After all saliva samples were completely dissolved, they were centrifuged at 1,610 × *g* for 15 min. The supernatant was used for this measurement. Saliva concentrations of IL-1β, IL-6, IL-10, IL-12p70, and TNF were measured using the BD Cytometric Bead Array (CBA) Human Inflammation Kit (BD Inc., California, USA). The assay procedures were performed following the manufacturer’s instructions, and data were analyzed using FCAP Array™ version 3.0 software (BD Inc., California, USA). PGE2 and vascular endothelial growth factor (VEGF) levels were also measured using a PGE2 enzyme-linked immunosorbent assay (ELISA) Kit (Enzo Life Science, Inc. New York, USA) and VEGF ELISA Kit (R&D Systems, Minneapolis, MN, USA), respectively, following the manufacturer’s instructions. All measurements were assayed in duplicate, and the readings were averaged. The outliers that exceeded the detection limit were excluded; IL-1β, IL-6, IL-10, IL-12p70, and TNF were 5,000 pg/mL, PGE2 was 2,500 pg/mL, and VEGF was 20,000 pg/mL.

### Statistical analysis

The comparison of the patient laboratory data and variables obtained by the questionnaire among the three groups, before TR (n = 18), OM (sampling points 2 and 3, n = 17), and after TR (n = 17), were analyzed using the Kruskal–Wallis test and Chi-square test. Statistical analyses of oral mucosal dryness and inflammatory mediators, comparing pre-index (n = 18) and post-index (n = 34) groups, comparing sampling points 1 and 4, were conducted using the Mann–Whitney U test. This grouping was adopted because OM is caused by cancer treatment which is the main intervention for the patients. The statistical differences in oral mucosal dryness and inflammatory mediator concentrations between the HV and the pre- and post-index groups were analyzed using Dunn’s multiple comparisons test. Oral mucosal dryness frequency of < 28 or ≥ 28 in HV versus the pre- and post-index groups was analyzed using the Chi-square test. The correlation between OM grade, oral mucosal dryness, and inflammatory mediator concentrations was analyzed using the Spearman’s r test. Mann-Whitney test was used for the statistical analyses of mucosal dryness and inflammatory mediators between leukemia and head and neck carcinoma, and between the pre-index and post-index values. The correlation between age and each HV mediator was performed using Spearman’s r test.

The obtained results were subjected to statistical analysis using GraphPad Prism 7 (GraphPad Software Inc., California, USA), and *p*-values < 0.05 were considered statistically significant.

## Results

### Patient characteristics

Eighteen patients (twelve with leukemia and six with head and neck carcinoma) were included in the analysis (ten men and eight women) (Table 1). All patients with leukemia received BMT or CTx; however, only two received RT. Six and five patients with leukemia were administered fludarabine/melphalan/methotrexate (FLU/MEL/MTX) and intravenous busulfan/cyclophosphamide/methotrexate (ivBU/CY/MTX), respectively. Two and three patients with head and neck carcinoma were administered C-mab and CDDP, respectively. RT was administered to four patients with head and neck carcinoma.

Four patients experienced grade 3 OM; four had grade 2 OM; six had grade 1 OM; and four did not experience OM (grade 0). We collected saliva from all patients at sampling points 1, and 17 saliva samples were taken at sampling points 2, 3 and 4 for 18 patients, with the remaining patient without OM excluded from sampling (Fig 1).

A dentist collected dental information before TR (Table 2). The PCR and BOP were 18% (range, 1–63%) and 11% (range, 4–60%), respectively. Except four patients who did not have records, all patients had teeth with pocket depths of > 4 mm. Tooth mobility was 0 in eight of fourteen patients. The median total number of teeth was 28. Periodontitis was determined by the maximum pocket depth. Five patients had severe periodontitis, and others had moderate periodontitis.

We collected the laboratory data at each sampling point and grouped the data regarding OM occurrence, improvement, and aggravation (S1 Table). White blood cell counts, platelet, hemoglobin, total protein (TP), and albumin (Alb) levels were significantly decreased by the TR ((*p* = 0.0099, *p* = 0.0038, *p* = 0.0028, *p* = 0.0004, *p* = 0.0002; respectively). C-reactive protein was significantly increased from 0.06 to 1.2 mg/dL (*p* = 0.0078). No significant differences was observed in neutrophil level among the groups.

We collected information on dietary intake times, sleep times, caffeine intake within 1 hour of sample collection, TPN, and dental treatment within 24 hours of sample collection, using the questionnaire administered at each sampling point (S2 Table). A significant difference was observed in the number of patients receiving TPN and dental treatment within 24 h in the pre-TR, OM, and post-TR groups (*p* < 0.0001 and *p* = 0.0491, respectively).

### Changes in oral mucosal dryness and inflammatory mediators

**Fig 2:**
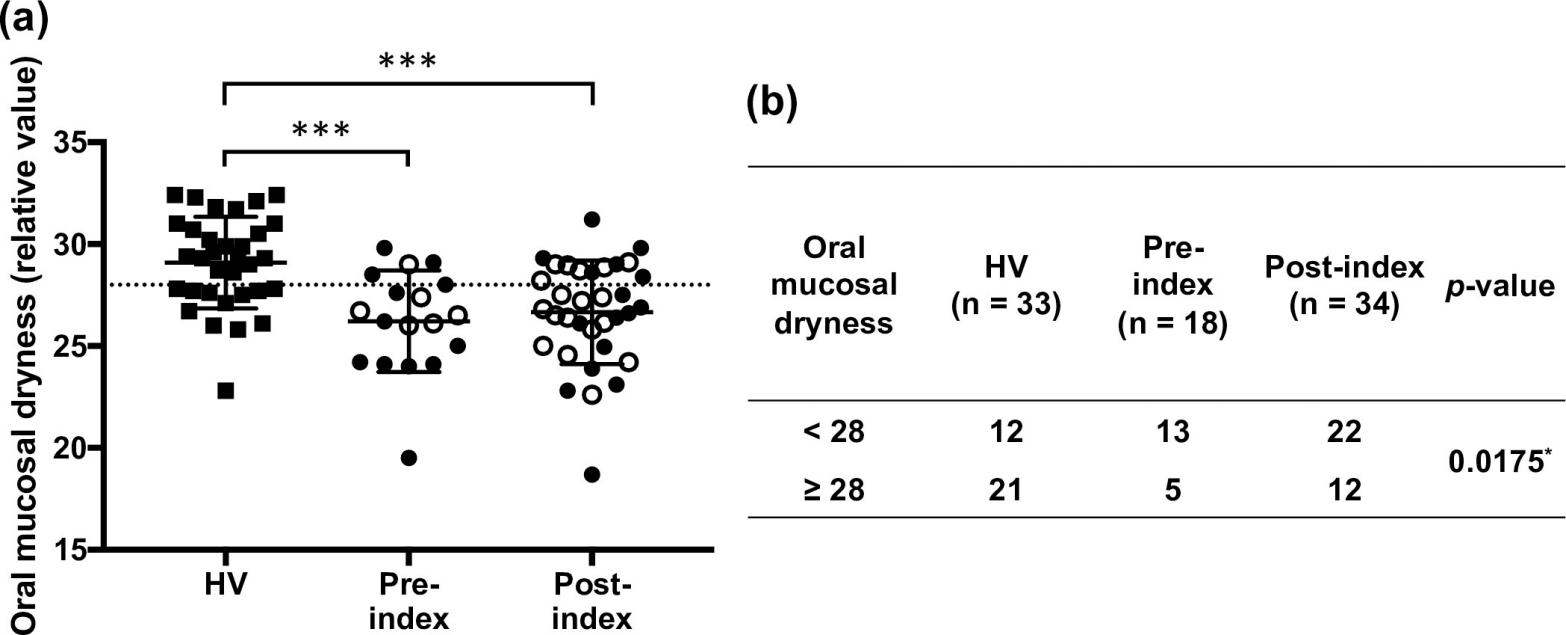
Comparison of oral mucosal dryness between HV (n = 33) and pre- and post-index patients with cancer (n = 18 and n = 34, respectively). All data for the post-index group are obtained at sampling points 2 within 3 days of an OM event, 3 if and when the OM event improved or worsened, and 4 within 7 days after treatment completion. (a) Data are shown as means ± standard deviations. The closed circles for the index groups represent patients with leukemia, while the open circles represent patients with head and neck carcinoma. Oral mucosal dryness has no unit. The dotted line shows the cut-off value for dryness (y = 28). Statistical differences between the HV and each index group are analyzed using Dunn’s multiple comparisons test (***p < 0.001). (b) Oral mucosal dryness is divided into two groups (< 28 and ≥ 28), and the frequency is analyzed using the Chi-square test (*p < 0.05). HV, healthy volunteers; OM, oral mucositis.

**Fig 3:**
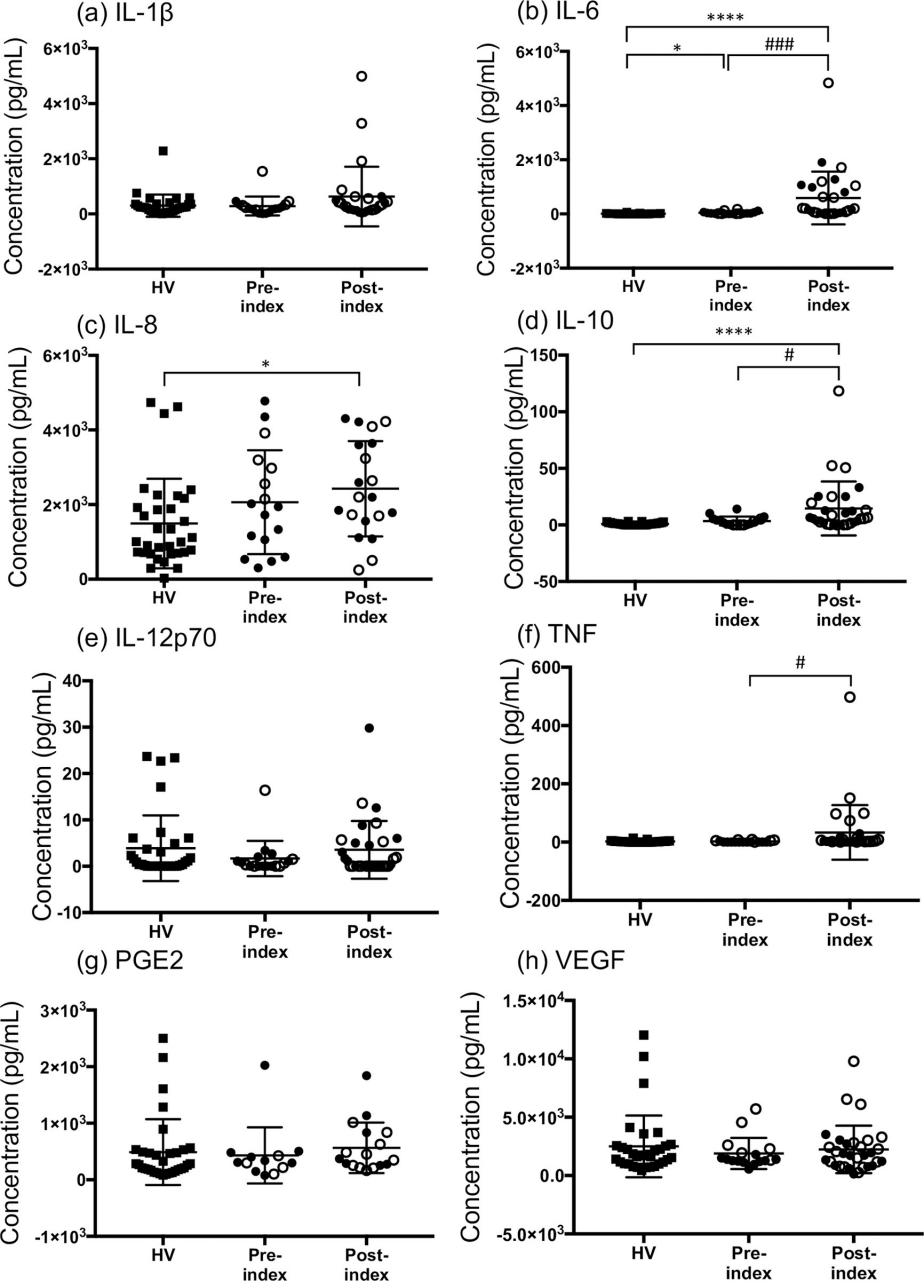
Concentration differences in salivary inflammatory mediators between healthy volunteers (HV) and pre- and post-index patients with cancer. (a) IL-1β, (b) IL-6, (c) IL-8, (d) IL-10, (e) IL-12p70, (f) TNF, (g) PGE2, and (h) VEGF. Data are shown as mean ± standard deviation. The closed circles for the index groups represent patients with leukemia, while the open circles represent patients with head and neck carcinoma. All data for the post-index group were obtained at sampling points 2, 3, and 4, as described in the methods section. The data in the pre-index group (IL-1β and VEGF; n = 1 and PGE2; n = 5) and the post-index group (IL-1β; n = 5, IL-6; n = 4, IL-8; n = 14, IL-10, IL-12p70, TNF, and VEGF; n = 3, and PGE2; n = 17) are missing because of low volume of saliva sampled or detection limits. The statistical differences between the HV and each index group are analyzed using Dunn’s multiple comparisons test (*p < 0.05 and ****p < 0.0001). The statistical differences between the pre- and post-index groups (Table 3) are analyzed using the Mann–Whitney U test (^#^*p* < 0.05 and ^###^*p* < 0.001).

Participants were divided into two groups based on the oral mucosal dryness and inflammatory mediator concentration data: the pre-index group (sampling point 1, n = 18) and the post-index group (sampling points 2, 3, and 4, n = 34) (Fig 1 and Online Resource 2). The median measurement of oral mucosal dryness was 26.4 (min-max, 19.5–29.8) and 26.9 (min-max, 18.7–31.2) in the pre- and post-index groups, respectively, and no significant difference was detected (*p* = 0.7014, Fig 2A). IL-6, IL-10, while TNF concentrations in the saliva of patients with cancer were significantly higher in the post-index group than in the pre-index group (*p* = 0.0002, Fig 3B; *p* = 0.0364, Fig 3D; and *p* = 0.0160, Fig 3F, respectively). However, no significant differences were detected in the concentrations of salivary IL-1β, IL-8, IL-12p70, PGE2, and VEGF among these groups (Table 3, Fig 3).

**Table 3:** Differences in measurements between pre- and post-index patients with cancer.

	Patients with cancer		*p*-value
	Pre-index (n = 18)	Post-index (n = 34)	
Oral mucosal dryness	26.4 (19.5–29.8)	26.9 (18.7–31.2)	0.7014
IL-1β (pg/mL)	196.8 (9.0–1,543.9)	294.2 (30.2–4,987.1)	0.2682
IL-6 (pg/mL)	18.2 (1.7–174.6)	163.7 (12.1–4,833.9)	0.0002^###^
IL-8 (pg/mL)	1,941.5 (304.7–4,776.9)	2,198.7 (249.1–4,308.3)	0.4065
IL-10 (pg/mL)	2.7 (0–14.4)	5.5 (0–118.4)	0.0364^#^
IL-12p70 (pg/mL)	0.5 (0–16.4)	0.4 (0–29.8)	0.5074
TNF (pg/mL)	0 (0–8.7)	2.5 (0–498.0)	0.0160^#^
PGE2 (pg/mL)	307.6 (76.7–2,024.3)	372.2 (164.7–1,841.3)	0.3411
VEGF (pg/mL)	1,396.8 (560.0–5,715.7)	1,736.0 (146.4–9,797.2)	0.7815

All measurements are described as medians (ranges). All data of the post-index group consist of the sampling points 2, 3, and 4, as shown in the Methods section. The data in the pre-index group (IL-1β and VEGF; n = 1 and PGE2; n = 5) and the post-index group (IL-1β; n = 5, IL-6; n = 4, IL-8; n = 14, IL-10, IL-12p70, TNF, and VEGF; n = 3, and PGE2; n = 17) are missing because of a low volume of saliva sampling or detection limits. The statistical differences between each measurement in pre- and post-index groups are analyzed using the Mann–Whitney U test (^#^*p* < 0.05, ^###^*p* < 0.001). Abbreviations: IL, interleukin; TNF, tumor necrosis factor; PGE2, prostaglandin E2; VEGF, vascular endothelial growth factor.

**Fig 4:**
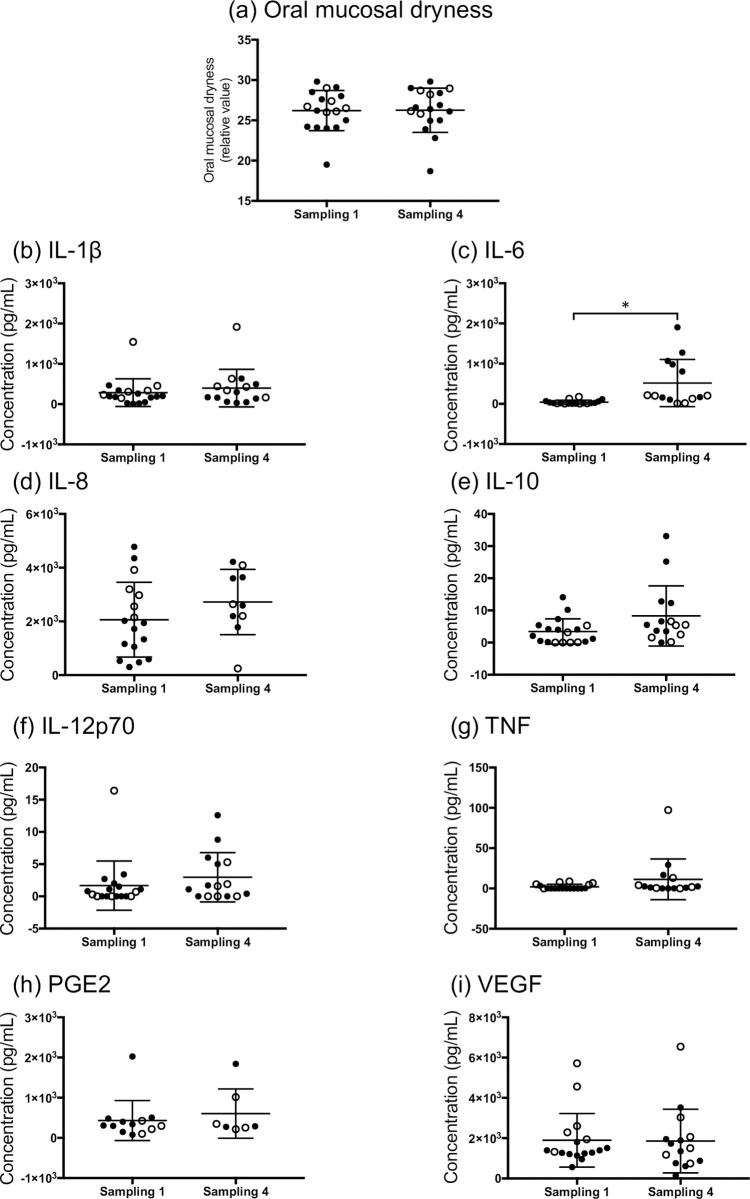
Concentration differences in oral mucosal dryness and salivary inflammatory mediators between sampling point 1 and 4. (a) Oral mucosal dryness, (b) IL-1β, (c) IL-6, (d) IL-8, (e) IL-10, (f) IL-12p70, (g) TNF, (h) PGE2, and (i) VEGF. Data are shown as mean ± standard deviation. The closed circles represent patients with leukemia, while the open circles represent patients with head and neck carcinoma. Sampling was done in group 1 and 4 before and after cancer treatment, as described in the methods section. The statistical differences between the sampled 1 and 4 groups were analyzed using the Mann–Whitney U test (*p < 0.05).

We also compared the value of each mediator between sampling point 1 (before TR) and 4 (after TR) (Fig 4). Only IL-6 salivary concentration was significantly increased in the sampling point 4 compared to point 1 (*p* < 0.0001, Fig 4C); however, there was no significant difference in other mediators between the sampling points.

### Oral mucosal dryness and inflammatory mediators in HV versus patients with cancer

We compared oral mucosal dryness and the concentration of inflammatory mediators in the HV group with those in the pre- and post-index groups. Oral mucosal dryness was significantly lower in the pre- and post-index groups than in HV (*p* < 0.001, Fig 2A). We divided the HV and patients into two groups based on oral mucosal dryness: the dry group, < 28, and the not-dry group, ≥ 28. A significant difference in the frequency of dryness was detected between HV and the pre- and post-index groups (*p* = 0.0175, Fig 2B). The salivary concentrations of IL-6, IL-8, and IL-10 were significantly higher in the post-index group than in HV (*p* < 0.0001, *p* < 0.05, *p* < 0.0001, respectively; Fig 3B–3D). No significant differences in other salivary mediators were observed between HV and the pre-index group. The correlation between age and each mediator was preliminarily determined in the HV group. There was significantly negative correlation between IL-12p70 and age (S4 Table), but none for oral mucosal dryness, IL-1β, IL-6, IL-10, TNF, PGE2, and VEGF.

### Correlation of OM grade with oral mucosal dryness and inflammatory mediators

**Fig 5:**
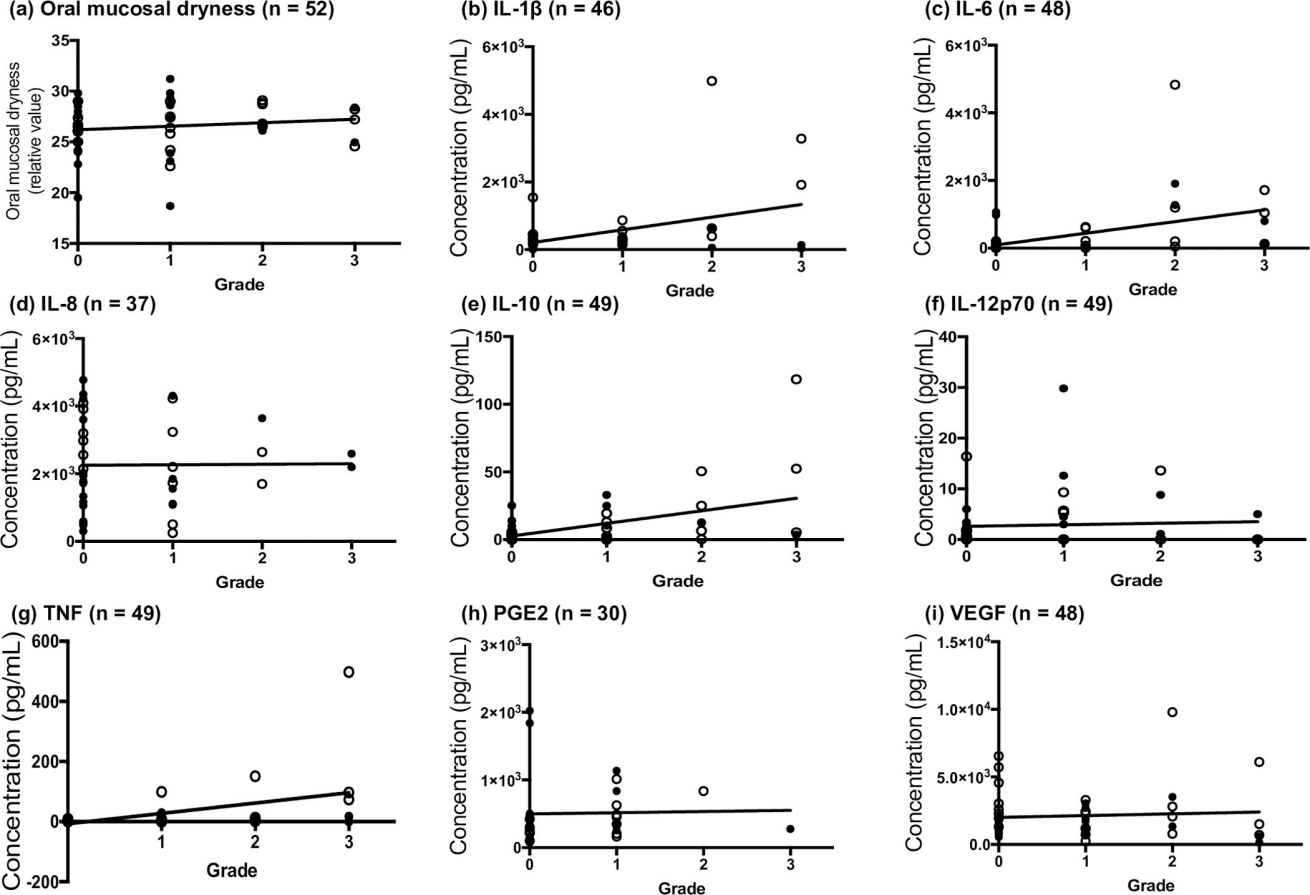
Correlation between the oral mucositis (OM) grade and the measurements. (a) Oral mucosal dryness and (b) IL-1β, (c) IL-6, (d) IL-8, (e) IL-10, (f) IL-12p70, (g) TNF, (h) PGE2, and (i) VEGF in the saliva of patients with cancer. The closed circles for the index groups represent patients with leukemia, while the open circles represent patients with head and neck carcinoma. The correlation of OM grade with each measurement is analyzed using Spearman’s r test.

**Fig 6:**
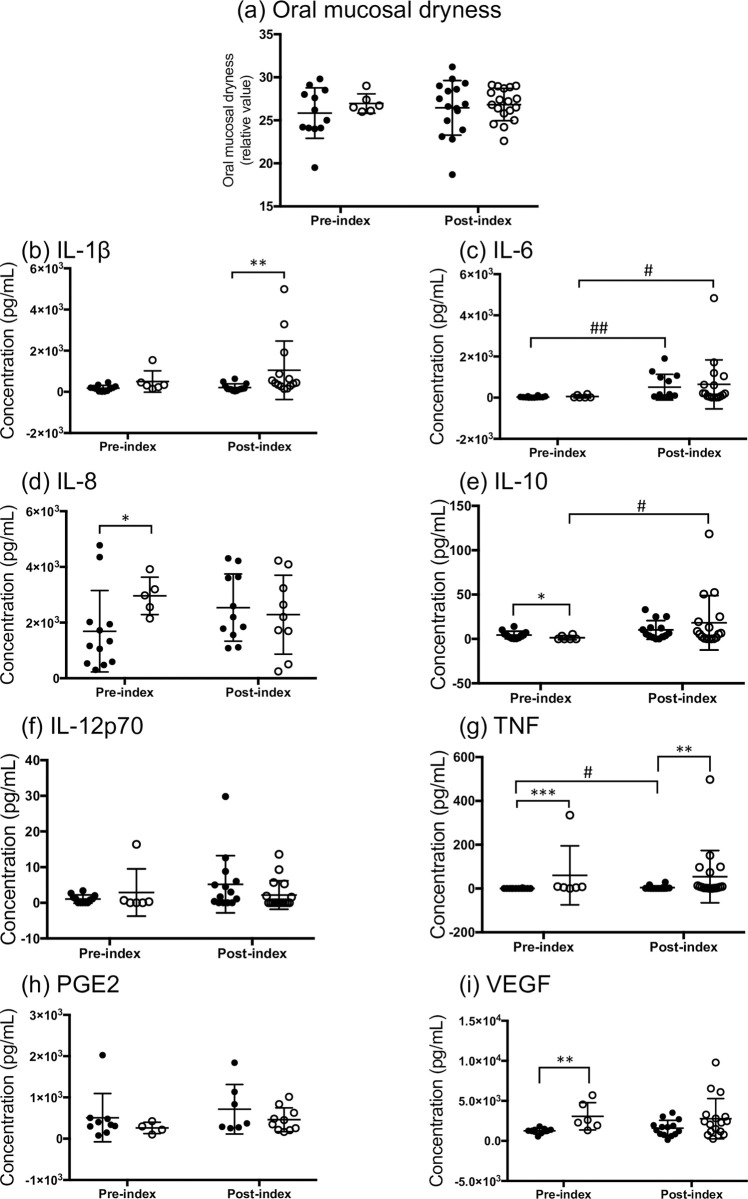
Concentration differences in salivary inflammatory mediators between pre- and post-index patients in each cancer group. (a) Oral mucosal dryness, (b) IL-1β, (c) IL-6, (d) IL-8, (e) IL-10, (f) IL-12p70, (g) TNF, (h) PGE2, and (i) VEGF. Data are shown as mean ± standard deviation. The closed circles represent patients with leukemia, while the open circles represent patients with head and neck carcinoma. All data for the post-index group were obtained at sampling points 2, 3, and 4, as described in the methods section. The statistical differences between the leukemia and head and neck carcinoma groups are analyzed using the Mann–Whitney U test (*p < 0.05, **p < 0.01, and ***p < 0.001). The statistical differences between the pre- and post-index groups in each cancer were analyzed using the Mann–Whitney U test (^#^*p* < 0.05, and ^##^*p* < 0.01).

We studied the correlation of OM grade and oral mucosal dryness with the concentration of inflammatory mediators in patient saliva samples. During the sampling schedule, there were 24 samples with grade 0, 16 with grade 1, 7 with grade 2, and 5 with grade 3. The salivary concentrations of IL-6, IL-10, and TNF were significantly positively correlated with OM grade (*p* = 0.0004, r = 0.4939; *p* = 0.0171, r = 0.3394; and *p* = 0007, r = 0.4662, respectively; Table 4 and Fig 6). Among these three mediators, only TNF concentration median was increased with increasing OM grade (Table 4 and Fig 5).

**Table 4:** Correlation between the oral mucositis grade and the measurements.

	OM				*p*-value	r
	Grade 0 (n = 24)	Grade 1 (n = 16)	Grade 2 (n = 7)	Grade 3 (n = 5)		
**Oral mucosal dryness**	26.3 (19.5–29.8)	27.5 (18.7–31.2)	26.9 (26.1–29.1)	27.2 (24.6–28.4)	0.2751	0.1542
**IL-1β (pg/mL)**	215.7 (9.0–1,543.9)	171.1 (105.1–866.9)	630.1 (58.3–4,987.1)	1,027.7 (30.2–3,283.1)	0.2742	0.1647
**IL-6 (pg/mL)**	23.0 (1.7–1,069.8)	45.3 (12.5–628.2)	1,235.8 (45.0–4,833.9)	804.3 (106.1–1,719.8)	0.0004***	0.4939
**IL-8 (pg/mL)**	2,024.4(304.7–4,776.9)	1,728.3 (249.1–4,308.3)	2,644.1 (1,696.9–3,642.0)	2,393.3 (2,195.1–2,591.4)	0.9407	0.01266
**IL-10 (pg/mL)**	3.6 (0–25.2)	2.5 (0–33.1)	12.5 (0.3–50.6)	5.4 (3.5–118.4)	0.0171*	0.3394
**IL-12p70 (pg/mL)**	0.8 (0–16.4)	1.5 (0–29.8)	0.6 (0–13.6)	0 (0–5.0)	0.7055	-0.05537
**TNF (pg/mL)**	0 (0–8.7)	2.2 (0–98.8)	5.7 (0.9–150.9)	73.8 (0–498.0)	0.0007***	0.4662
**PGE2 (pg/mL)**	299.8 (76.7–2,024)	452.5 (164.7–1,135.5)	837.0 (837.0–837.0)	275.3 (275.3–275.3)	0.2091	0.2361
**VEGF (pg/mL)**	1,397.9 (560.0–6,544.6)	1,843.4 (258.8–3,288.5)	2,435.7 (790.2–9,797.2)	707.0 (146.4–6,111.4)	0.8803	-0.02233

All measurements are described as median (range). The correlations of OM grade with each measurement are analyzed using Spearman’s r test (*p < 0.05, ***p < 0.001). Abbreviations: IL, interleukin; TNF, tumor necrosis factor; PGE2, prostaglandin E2; VEGF, vascular endothelial growth factor; OM, oral mucositis.

### Comparison of oral mucosal dryness and inflammatory mediators between leukemia and head and neck carcinoma

We compared inflammatory mediators between leukemia and head and neck carcinoma (Fig 6). There were significant differences in IL-8, IL-10, TNF, and VEGF concentration between leukemia and head and neck carcinoma at pre-index (*p* < 0.05, *p* < 0.05, *p* < 0.001, and *p* < 0.01, respectively; Fig 6D, 6E, 6G and 6I) and IL-8, TNF, VEGF concentration were higher in the saliva of head and neck carcinoma patients than leukemia patients. On the other hand, IL-10 concentration of leukemia patients was higher than those of head and neck carcinoma patients. In the post-index group, the concentration of IL-1β and TNF in head and neck carcinoma were significantly higher than those in leukemia (*p* < 0.01 and *p* < 0.01, respectively; Fig 6B and 6G). Next, the mediator concentration between pre-index and post-index were compared in each cancer group. Only IL-6 concentration increased significantly in both leukemia and head and neck carcinoma groups (*p* < 0.01 and *p* < 0.05, respectively; Fig 6C). In the leukemia group, TNF concentration was higher in the post-index group, compared with those in the pre-index group (*p* < 0.05; Fig 6G), and IL-10 concentration was significantly higher in the post-index group of the head and neck carcinoma group, compared to the pre-index group (*p* < 0.05; Fig 6E). There was no significant difference in oral mucosal dryness, IL-12p70, and PGE2 (Fig 6A, 6F and 6H).

## Discussion

We measured the concentration of inflammatory mediators in the saliva and evaluated the oral mucosal dryness in patients with cancer to determine a candidate variable potentially associated with OM. Additionally, we evaluated the relationship of OM and oral mucosal dryness with inflammatory mediators, and dental and laboratory data in patients with cancer compared to those in HV. The results showed that IL-6, IL-10, and TNF concentrations in the saliva of patients with cancer were significantly higher in the post-index group than in the pre-index group and significantly positively correlated with OM grade. Moreover, the salivary concentrations of IL-6, IL-8, and IL-10 were significantly higher in the post-index group than in the HV group. Additionally, regardless of measuring before or after CTx and RT, oral mucosal dryness was observed significantly more frequently in the patients with cancer than in the HV group.

Previous studies have shown that several mediators may affect the occurrence of OM in situ [14, 15]; however, to clinically understand OM, a non-invasive and easily collectable sample should be used. Additionally, a comprehensive understanding of salivary inflammatory mediators in OM is lacking. Thus, we comprehensively measured representative inflammatory mediators, including IL-1β, IL-6, IL-10, IL-12p70, TNF, PGE2, and VEGF concentrations in the saliva of patients with cancer, which is convenient to measure, less invasive to collect, and can be done repeatedly. Saliva is widely used as patient sample in the analysis for understanding diseases, pathology, monitoring, and preventive medicine [16–18]. These findings suggest potential advantage in using saliva, compared to using blood, cell, and tissues, which require invasion.

Numerous studies focus on individual cancer types; thus, it is difficult to generalize OM as a common side effect of therapy in patients with cancer [6, 8, 15, 19]. We recruited several patients with leukemia and head and neck carcinoma in an attempt to provide a broader representation of patients with OM. Moreover, most studies include either patients with cancer or HV [20], and only a few studies have compared between the saliva samples in HV and patients with cancer in the relation with oral mucosal dryness. Furhtermore, in the studies that do compare the groups, OM grade changes throughout the course of cancer TR have not been reported [21]. Although health care professionals should assess oral mucosal dryness in patients with advanced disease [11, 22–24], it has not been clarified when and how they need to assess it to evaluate the oral condition and to monitor OM. While inflammatory mediators and oral mucosal dryness may be important factors in OM [25, 26], no research has been conducted to measure these parameters in relation to OM simultaneously to evaluate their association with OM grade. Therefore, we addressed the current limitations in this field by comparing the relationships between OM and salivary variables.

Here, OM occurred in fourteen of the eighteen patients with cancer (approximately 78%) in the post-index group. Four patients (29%) had a maximum OM grade of 3 during the study period. All patients with head and neck carcinoma had OM in our study. A previous study reported that the majority of patients with head and neck carcinoma (83%) developed OM (mild, 19%; moderate, 35%; and severe, 28%) [8]. OM occurrence in patients who have undergone BMT has been reported in 76.3% [27] and 99% [28] of treated individuals. Our cohort of patients showed a higher rate of OM occurrence and stage than those in the previous report. Notably, the patient without OM had received CTx or RT during the study period; therefore, OM may have occurred after the study period since the period lasted only a week post-TR.

We collected the patient dental information before TR initiation to assess their dental condition (Table 2). PCR was used to evaluate oral hygiene [29]; a higher score indicated a higher risk of periodontitis. Additionally, we determined the frequency of maintenance using BOP. Patients who presented with high BOP (> 16% of possible sites) presented increased attachment loss [30]. Here, the median PCR and BOP were 9.8% and 10.5%, respectively. The oral care support teams assessed patients regularly, and this intervention may have affected patient oral conditions during hospitalization. However, all patients had teeth with pocket depths of more than 4 mm except for missing data from four patients. The severity of periodontitis was assessed as moderate or severe before cancer TR onset. The value of inflammatory mediators and oral mucosal dryness measured in this study might have been affected by the patients’ oral condition.

After saliva sample collection, we obtained several variables potentially affecting the concentration of immune mediator secretion (S2 Table). Only the number of patients receiving TPN was significantly increased and the number of patients who had dental treatment within 24 h was significantly decreased following CTx and RT administration. These results suggested that patients who received CTx and RT tend to be unable to tolerate oral intakes; thus, the need to receive oral supportive care might be decreased. Cancer TR causes appetite loss and induces switching to TPN, potentially explaining the significantly decreased TP and Alb levels (S1 Table).

Oral mucosal dryness was compared between the pre- and post-index groups, and no significant difference was observed (Table 3). However, in the pre- and post-index groups, the median of oral mucosal dryness was 26.4 and 26.9, respectively, with 28 being the cut-off value used to define oral dryness [31]. Moreover, when compared with HV, both the pre-and post-index groups showed significantly less oral mucosal dryness, and the number of individuals with oral dryness was higher in the patient groups (Fig 2). This result is a novel observation and suggests that active interventions by the oral care support team are required to maintain patient oral moisture, even before cancer TR onset.

The concentrations of IL-6, IL-10, and TNF in the patient saliva were increased significantly post-index compared to those pre-index (Table 3). Additionally, compared with before TR, the salivary IL-6 concentration was significantly increased after TR (Fig 4). A systematic review of 34 studies showed that IL-6 and TNF were associated with the severity of oral mucosal tissue damage in patients with cancer; the most investigated cytokines were IL-6, TNF, and IL-10 [32]. Therefore, our results are consistent with previous research. Moreover, IL-6, IL-8, and IL-10 concentrations in the post-index patient saliva samples were significantly higher than those of HV (Fig 3). These mediators were reported to be related to OM pathology [9, 33]. The outliers of IL-6, IL-10 and TNF in the post-index group were detected in saliva samples from different patients with head and neck carcinoma, and all sampling time was at OM occurrence. This result suggested that the oral inflammation by carcinoma itself might be caused by the increase of these mediators.

Significant positive correlations were recorded between OM grade and IL-6, IL-10, and TNF concentrations in the saliva of the post-index group (Fig 5). A previous study that detected salivary cytokines in patients with head and neck cancer with concurrent CTx and RT reported a significant increase in the salivary levels of IL-1β, IL-6, and TNF, that were all positively associated with the mucosal toxicity severity [10]. We included patients with head and neck carcinoma; thus, our results may be similar to those in a previous report.

Lastly, we compared the changes in oral mucosal dryness and inflammatory mediator concentrations between leukemia and head and neck carcinoma. The head and neck carcinoma group had significantly higher concentrations of IL-8, TNF, and VEGF in the pre-index group, and IL-1β and TNF in the post-index group. Because oral inflammation had occurred in advance of OM, due to cancer progression in head and neck carcinoma patients, these mediators could have been detected to be higher compared to those in leukemia patients. IL-10 and TNF concentrations were significantly higher in the post-index group compared to the pre-index group among head and neck carcinoma and leukemia patients, respectively. Regardless of cancer type, only IL-6 concentration significantly increased in the post-index group. Based on our results and previous findings, we suggest that salivary IL-6, IL-10, and TNF concentrations are potential markers associated with the onset and severity of OM [9, 32].

This study has several limitations. First, the saliva volume was limited; therefore, we could not perform oxylipins measurement. Salivary oxylipins are informative mediators for understanding the regulation of inflammation [34–37]; thus, the detection of these mediators should be conducted in further study. Additionally, high mediator production may have occurred in patients with high severity of periodontal disease or head and neck carcinoma. Thus, further studies including more patients with various cancer types are required. Furthermore, all patients received different kinds of anticancer drugs and radiation regimens; thus, it was impossible to integrate the cancer TR type into our analysis. Second, the volume of saliva differed between the patients and sampling points, resulting in missing data for some mediator measurements. Third, we could not evaluate the preventive effects of the medication for OM and the value of professional intervention because all patients were administered preventive medication for OM and had been followed up by the oral health care support team. These medications were administrated as single doses to patients as preventive care for OM; also, we asked patients to avoid using them within 1 h before sampling. Thus, we stated that the effects on saliva PH, composition, and flow due to these medications would be less. In this study, it was difficult to obtain patients ethically because most of them were supposed to experience OM during the study period. Furthermore, we did not consider the use of medications such as non-steroidal anti-inflammatory drugs and opioids, which potentially could affect inflammatory mediator secretion.

In conclusion, this pilot study used the saliva collected from patients with cancer and HV to identify candidate biomarkers, which changed according with the OM severity and outcomes. Additionally, we measured the relationships of OM and oral mucosal dryness with inflammatory mediators, and dental and laboratory data in patients with cancer and compared it with those in HV. We identified salivary IL-6, IL-10, and TNF as potential biomarkers related to OM; they showed a significant relationship with OM occurrence and grade. Moreover, oral mucosal dryness in patients with cancer, even before CTx and RT onset, was significantly lower than that in HV.

## Supporting information

S1 Table Laboratory data of patients at each sampling point. (DOCX)

S2 Table Summary of patient questionnaire results at each sampling point. (DOCX)

S3 Table Characteristics of healthy volunteers. (DOCX)

S4 Table Relationship between age and mediators in healthy volunteer. (DOCX)

S5 Table The minimal data set. (DOCX)
