# Supplementary material for: Salivary inflammatory mediators as biomarkers for oral mucositis and oral mucosal dryness in cancer patients: A pilot study
Source: PLoS One. 2022 Apr 27;17(4):e0267092. doi: 10.1371/journal.pone.0267092 (PMC9045655; doi:10.1371/journal.pone.0267092)
Supplement: S1 Table — (DOCX) [file pone.0267092.s001.docx]

**Supporting information**

Salivary inflammatory mediators as biomarkers for oral mucositis and oral mucosal dryness in cancer patients: A pilot study

Anna Kiyomi^1*^, Kensuke Yoshida^2,3^, Chie Arai^1^, Risa Usuki^1^, Kyosuke Yamazaki^1^, Naoto Hoshino^3^, Akira Kurokawa^2^, Shinobu Imai^1^, Naoto Suzuki^3^, Akira Toyama^3^, and Munetoshi Sugiura^1^

* Corresponding author: Dr. Anna Kiyomi

E-mail: akiyomi@toyaku.ac.jp

**S1 Table.** **Laboratory data of patients at each sampling point.**

|  | **Pre TR (n = 18)** | **OM (n = 17)** | **Post TR (n = 17)** | ***p*-value** |
| --- | --- | --- | --- | --- |
| WBC (/mL) | 3830 (840–5720) | 3600 (90–6770) | 60 (10–9060) | 0.0099^*^ |
| PLT (× 10^4^/mL) | 19.45 (1.6–32.4) | 17.75 (5.2–26.7) | 3.3 (0.7–23.1) | 0.0038^*^ |
| Neu (× 10^3^/mL) | 2.16 (0.17–3.52) | 2.81 (1.02–5.99) | 2.67 (0.07–7.69) | 0.3220 |
| Hb (g/dL) | 11.5 (8.4–14.6) | 12.4 (9.2–15.2) | 8.6 (6.0–14.1) | 0.0028^*^ |
| TP (g/dL) | 6.8 (6.2–7.8) | 6.8 (5.9–7.4) | 6.0 (5.4–7.0) | 0.0004^*^ |
| Alb (g/dL) | 4.1 (3.3–4.6) | 3.6 (2.5–4.1) | 3.2 (2.5–4.1) | 0.0002^*^ |
| CRP (mg/dL) | 0.06 (0.02–0.84) | 0.15 (0.02–0.88) | 1.2 (0.03–15.93) | 0.0078^*^ |

All variables are presented as median (range). The OM group contained sampling points 2 and 3. Statistical differences between the groups are analyzed using the Kruskal–Wallis test (**p* < 0.05).

Abbreviations: TR, treatment; OM, oral mucositis; WBC, white blood cell; PLT, platelet; Neu, neutrophil; Hb, hemoglobin; TP, total protein; Alb, albumin; CRP, C-reactive protein.
