# Supplementary material for: Salivary inflammatory mediators as biomarkers for oral mucositis and oral mucosal dryness in cancer patients: A pilot study
Source: PLoS One. 2022 Apr 27;17(4):e0267092. doi: 10.1371/journal.pone.0267092 (PMC9045655; doi:10.1371/journal.pone.0267092)
Supplement: S2 Table — (DOCX) [file pone.0267092.s002.docx]

**Supporting information**

Salivary inflammatory mediators as biomarkers for oral mucositis and oral mucosal dryness in cancer patients: A pilot study

Anna Kiyomi^1*^, Kensuke Yoshida^2,3^, Chie Arai^1^, Risa Usuki^1^, Kyosuke Yamazaki^1^, Naoto Hoshino^3^, Akira Kurokawa^2^, Shinobu Imai^1^, Naoto Suzuki^3^, Akira Toyama^3^, and Munetoshi Sugiura^1^

* Corresponding author: Dr. Anna Kiyomi

E-mail: akiyomi@toyaku.ac.jp

**S2 Table.** **Summary of the patient questionnaire results at each sampling point.**

|  | **Pre TR (n = 18)** | **OM (n = 17)** | **Post TR (n = 17)** | ***p*-value** |
| --- | --- | --- | --- | --- |
| Dietary intake time  (hours before, mean ± SD) | 3.2 ± 0.9 | 3.0 ± 0.9^a^ | 6.6 ± 9.5^a^ | 0.3924 |
| Sleep time (hours, mean ± SD) | 6.9 ± 1.9 | 6.4 ± 2.5^b^ | 5.7 ± 2.5 | 0.3767 |
| Caffeine intake within 1 hour | 2/18 | 2/17 | 0/17 | 0.3482 |
| TPN | 0/18 | 0/17 | 9/17 | < 0.0001^*^ |
| Dental treatment  within 24 hours | 5/18 | 5/17 | 0/17 | 0.0491^*^ |

^a^Two patient data are missing, and ^b^one patient data are missing. The OM group contains sampling points 2 and 3. The statistical differences in dietary intake time and sleep time among groups are analyzed using the Kruskal–Wallis test. The statistical differences in caffeine intake, TPN, and dental treatment among groups are analyzed using the chi-square test (**p* < 0.05).

Abbreviations: TR, treatment; OM, oral mucositis; SD, standard deviation; TPN, total parenteral nutrition.
