# Supplementary material for: Salivary inflammatory mediators as biomarkers for oral mucositis and oral mucosal dryness in cancer patients: A pilot study
Source: PLoS One. 2022 Apr 27;17(4):e0267092. doi: 10.1371/journal.pone.0267092 (PMC9045655; doi:10.1371/journal.pone.0267092)
Supplement: S3 Table — (DOCX) [file pone.0267092.s003.docx]

**Supporting information**

Salivary inflammatory mediators as biomarkers for oral mucositis and oral mucosal dryness in cancer patients: A pilot study

Anna Kiyomi^1*^, Kensuke Yoshida^2,3^, Chie Arai^1^, Risa Usuki^1^, Kyosuke Yamazaki^1^, Naoto Hoshino^3^, Akira Kurokawa^2^, Shinobu Imai^1^, Naoto Suzuki^3^, Akira Toyama^3^, and Munetoshi Sugiura^1^

* Corresponding author: Dr. Anna Kiyomi

E-mail: akiyomi@toyaku.ac.jp

**S3 Table.** **Characteristics of healthy volunteers.**

|  | **Total** |
| --- | --- |
| Age (years) | 24 (18–65) |
| Time asleep (hours) | 6 (3–18) |
| Oral mucosal dryness | 29.3 (22.8–32.4) |
| IL-1β (pg/mL) | 234.7 (3.6–2,285.1) |
| IL-6 (pg/mL) | 5.7 (0–71.1) |
| IL-8 (pg/mL) | 1,009.9 (27.6–4,735.5) |
| IL-10 (pg/mL) | 0.8 (0–3.3) |
| IL-12p70 (pg/mL) | 0.4 (0–23.7) |
| TNF (pg/mL) | 1 (0–13.7) |
| PGE2 (pg/mL) | 268.3 (82.0–2,500) |
| VEGF (pg/mL) | 1,710.2 (445.7–1,2048.1) |

All variables are presented as median (range).

Abbreviations: IL, interleukin; TNF, tumor necrosis factor; PGE2, prostaglandin E2; VEGF, vascular endothelial growth factor.
