# Supplementary material for: Salivary inflammatory mediators as biomarkers for oral mucositis and oral mucosal dryness in cancer patients: A pilot study
Source: PLoS One. 2022 Apr 27;17(4):e0267092. doi: 10.1371/journal.pone.0267092 (PMC9045655; doi:10.1371/journal.pone.0267092)
Supplement: S4 Table — (DOCX) [file pone.0267092.s004.docx]

**Supporting information**

Salivary inflammatory mediators as biomarkers for oral mucositis and oral mucosal dryness in cancer patients: A pilot study

Anna Kiyomi^1*^, Kensuke Yoshida^2,3^, Chie Arai^1^, Risa Usuki^1^, Kyosuke Yamazaki^1^, Naoto Hoshino^3^, Akira Kurokawa^2^, Shinobu Imai^1^, Naoto Suzuki^3^, Akira Toyama^3^, and Munetoshi Sugiura^1^

* Corresponding author: Dr. Anna Kiyomi

E-mail: akiyomi@toyaku.ac.jp

**S4 Table. Relationship between age and mediators in healthy volunteers.**

|  | **Correlation coefficient (r)** | ***p*-value** |
| --- | --- | --- |
| Oral mucosal dryness | 0.0191 | 0.9160 |
| IL-1β (pg/mL) | 0.1988 | 0.2673 |
| IL-6 (pg/mL) | 0.205 | 0.2524 |
| IL-8 (pg/mL) | 0.1783 | 0.3208 |
| IL-10 (pg/mL) | 0.09461 | 0.6005 |
| IL-12p70 (pg/mL) | -0.3617 | 0.0386^*^ |
| TNF (pg/mL) | 0.04355 | 0.8098 |
| PGE2 (pg/mL) | -0.09287 | 0.6132 |
| VEGF (pg/mL) | 0.1033 | 0.5673 |

The statistical correlation between age and each mediator are analyzed using the Spearman’s r test (**p* < 0.05).
