# Supplementary material for: Salivary inflammatory mediators as biomarkers for oral mucositis and oral mucosal dryness in cancer patients: A pilot study
Source: PLoS One. 2022 Apr 27;17(4):e0267092. doi: 10.1371/journal.pone.0267092 (PMC9045655; doi:10.1371/journal.pone.0267092)
Supplement: S5 Table — (DOCX) [file pone.0267092.s005.docx]

**Supporting information**

Salivary inflammatory mediators as biomarkers for oral mucositis and oral mucosal dryness in cancer patients: A pilot study

Anna Kiyomi^1*^, Kensuke Yoshida^2,3^, Chie Arai^1^, Risa Usuki^1^, Kyosuke Yamazaki^1^, Naoto Hoshino^3^, Akira Kurokawa^2^, Shinobu Imai^1^, Naoto Suzuki^3^, Akira Toyama^3^, and Munetoshi Sugiura^1^

* Corresponding author: Dr. Anna Kiyomi

E-mail: akiyomi@toyaku.ac.jp

**S5 Table. The minimal data set.**

| Cancer type | Timing | OM  grade | Oral mucosal dryness | IL-1β (pg/mL) | IL-6 (pg/mL) | IL-8 (pg/mL) | IL-10 (pg/mL) | IL-12p70 (pg/mL) | TNF (pg/mL) | PGE2 (pg/mL) | VEGF (pg/mL) |
| --- | --- | --- | --- | --- | --- | --- | --- | --- | --- | --- | --- |
| Leukemia | Before TR | 0 | 19.5 | 49.7 | 1.7 | 304.7 | 4.2 | 0.0 | 0.0 | - | 560.0 |
| Leukemia | Before TR | 0 | 25.0 | 465.8 | 16.4 | 4352.3 | 2.1 | 0.0 | 0.0 | - | 1808.7 |
| Leukemia | Before TR | 0 | 29.8 | 9.0 | 3.4 | 479.5 | 0.5 | 0.0 | 0.0 | 148.1 | 959.9 |
| Leukemia | Before TR | 0 | 29.1 | 200.0 | 16.1 | 1331.8 | 1.2 | 0.0 | 0.0 | 402.7 | 1209.6 |
| Leukemia | Before TR | 0 | 24.1 | 193.5 | 62.0 | 594.0 | 0.3 | 0.0 | 0.0 | 482.2 | 1271.6 |
| Leukemia | Before TR | 0 | 26.2 | 164.0 | 25.02 | 530.6 | 0.2 | 1.5 | 0.0 | 76.7 | - |
| Leukemia | Before TR | 0 | 27.6 | 179.5 | 114.0 | 2024.4 | 10.2 | 1.1 | 3.4 | 336.8 | 1283.3 |
| Leukemia | Before TR | 0 | 28.5 | 19.7 | 69.1 | 4776.9 | 14.1 | 2.7 | 0.0 | 2024.3 | 1396.8 |
| Leukemia | Before TR | 0 | 28 | 342.5 | 21.0 | 1057.4 | 5.4 | 1.1 | 0.0 | 299.7 | 1509.4 |
| Leukemia | Before TR | 0 | 24.2 | 188.0 | 5.7 | 1164.9 | 4.0 | 0.8 | 0.0 | - | 1134.8 |
| Leukemia | Before TR | 0 | 24.1 | 261.9 | 36.5 | 1941.5 | 4.1 | 2.0 | 0.4 | 504.8 | 1397.9 |
| Leukemia | Before TR | 0 | 24 | 24.2 | 20.0 | 1720.4 | 7.3 | 3.4 | 0.0 | 307.6 | 1240.7 |
| Leukemia | OM | 1 | 23.1 | 402.1 | 45.3 | 1558.2 | 10.4 | 0.0 | 0.0 | - | 836.4 |
| Leukemia | OM | 1 | 31.2 | 171.1 | 23.9 | 4308.3 | 1.9 | 4.5 | 0.8 | 256.6 | 1736.0 |
| Leukemia | OM | 1 | 28.6 | 105.1 | 14.9 | 1112.6 | 0.0 | 3.0 | 0.0 | 372.2 | 1232.3 |
| Leukemia | OM | 1 | 29.3 | 130.8 | 33.7 | 1846.7 | 25.0 | 29.8 | 3.6 | 1135.5 | 2706.0 |
| Leukemia | OM | 1 | 27.5 | 137.8 | 59.3 | 1080.6 | 2.4 | 0.0 | 0.0 | 836.1 | 3038.7 |
| Leukemia | After TR | 0 | 26.4 | 494.5 | 167.6 | 3602.6 | 5.5 | 0.0 | 0.0 | - | 879.7 |
| Leukemia | After TR | 0 | 22.8 | 46.9 | 1069.8 | - | 6.6 | 6.0 | 2.6 | 286.9 | 754.0 |
| Leukemia | After TR | 0 | 29 | 298.7 | 159.8 | 1780.3 | 0.0 | 0.4 | 0.0 | - | 1891.5 |
| Leukemia | After TR | 0 | 26.6 | 170.1 | 982.7 | 4218.8 | 25.2 | 1.7 | 1.3 | 1841.3 | 1728.9 |
| Leukemia | After TR | 1 | 18.7 | - | - | - | - | - | -- | - | - |
| Leukemia | After TR | 1 | 23.9 | - | - | - | - | - | - | - | - |
| Leukemia | After TR | 1 | 29.8 | 160.9 | - | - | 33.1 | 12.6 | 29.2 | - | 1950.7 |
| Leukemia | After TR | 2 | 26.9 | 58.3 | 1905.3 | 3642.0 | 12.3 | 1.1 | 2.5 | - | 1346.7 |
| Leukemia | After TR | 2 | 26.1 | 634.2 | 1275.3 | - | 12.8 | 8.8 | 0.9 | - | 3522.2 |
| Leukemia | After TR | 3 | 28.4 | 138.0 | 106.1 | 2591.4 | 3.5 | 0.0 | 0.0 | - | 608.1 |
| Leukemia | After TR | 3 | 24.95 | 30.2 | 804.3 | 2195.1 | 3.7 | 5.0 | 16.7 | 275.3 | 146.4 |
| Carcinoma | Before TR | 0 | 26.7 | 231.4 | 14.0 | 3918.9 | 0 | 0 | 0.0 | 101.1 | 1321.7 |
| Carcinoma | Before TR | 0 | 26.5 | 309.6 | 7.7 | 2147.8 | 3.2 | 0 | 7.9 | - | 5715.7 |
| Carcinoma | Before TR | 0 | 26 | 1543.9 | 127.1 | - | 0 | 0.3 | 8.7 | - | 2289.7 |
| Carcinoma | Before TR | 0 | 29 | 153.2 | 6.9 | 2977.6 | 0 | 0 | 5.2 | 299.8 | 1945.8 |
| Carcinoma | Before TR | 0 | 27.4 | 456.2 | 174.6 | 2558.2 | 5.3 | 16.4 | 4.6 | 424.1 | 4564.2 |
| Carcinoma | Before TR | 0 | 26.1 | 335.2 | 13.4 | 3198.2 | 0.1 | 0.7 | 6.8 | 217.9 | 2601.3 |
| Carcinoma | OM | 1 | 27.5 | 264.6 | 628.2 | 3239.9 | 8.6 | 5.7 | 3.2 | 627.4 | 3288.5 |
| Carcinoma | OM | 1 | 26.4 | 866.9 | 600.1 | 4227.2 | 13.1 | 9.3 | 6.1 | 164.7 | 2280.3 |
| Carcinoma | OM | 2 | 29.1 | - | 4833.9 | - | 25.1 | 13.6 | 9.0 | - | 9797.2 |
| Carcinoma | OM | 1 | 27.4 | 559.7 | 72.0 | 1728.3 | 0.7 | 0 | 9.3 | 486.4 | 1190.4 |
| Carcinoma | OM | 1 | 24.2 | 144.7 | 12.9 | - | 0 | 0 | 2.3 | 206.9 | 2428.7 |
| Carcinoma | OM | 1 | 29 | - | 73.7 | - | 19.4 | 0 | 98.8 | - | 258.8 |
| Carcinoma | OM | 1 | 22.6 | 289.7 | 12.5 | 502.3 | 0 | 0 | 2.1 | 452.5 | 2457.2 |
| Carcinoma | OM | 2 | 26.8 | 4987.1 | 1196.4 | - | 50.6 | 0 | 150.9 | - | 2807.1 |
| Carcinoma | OM | 2 | 28.85 | 398.2 | 45.0 | 1696.9 | 0.3 | 0 | 1.5 | 837.0 | 790.2 |
| Carcinoma | OM | 2 | 26.5 | - | - | - | - | - | - | - | - |
| Carcinoma | OM | 3 | 27.2 | 3283.1 | 1045.3 | - | 118.4 | 0 | 73.8 | - | 6111.4 |
| Carcinoma | OM | 3 | 24.55 | - | 1719.8 | - | 52.4 | 0 | 497.9 | - | 707.0 |
| Carcinoma | After TR | 0 | 25 | 427.8 | 12.1 | 4093.1 | 1.6 | 1.9 | 1.8 | 254.8 | 6544.6 |
| Carcinoma | After TR | 0 | 26.15 | 441.2 | 205.3 | - | 5.5 | 1.6 | 4.0 | 215.7 | 3031.5 |
| Carcinoma | After TR | 1 | 25.8 | 164.4 | 17.0 | 249.1 | 0.2 | 0 | 0.6 | 347.8 | 740.7 |
| Carcinoma | After TR | 1 | 28.95 | 341.5 | 214.4 | 2202.3 | 2.5 | 5.3 | 0.0 | 1014.1 | 1175.7 |
| Carcinoma | After TR | 2 | 28.7 | 630.1 | 200.5 | 2644.1 | 6.6 | 0 | 13.0 | - | 2064.4 |
| Carcinoma | After TR | 3 | 28.2 | 1917.4 | 129.6 | - | 5.4 | 0 | 97.3 | - | 1501.1 |

The raw data detected in this study.
